# A behavior change wheel-based interactive pictorial health education program for hypertensive patients with low blood pressure health literacy: study protocol for a randomized controlled trial

**DOI:** 10.1186/s13063-022-06300-1

**Published:** 2022-05-03

**Authors:** Wei Gan, Qinghua Zhang, Dan Yang, Jinyu Yin, Yujie Wang, Li Song, Ting Chen, Huan Qi

**Affiliations:** 1 School of Medicine & Nursing Sciences, HuZhou University, Huzhou, Zhejiang China; 2 Present address: Department of Nursing, Jiangxi Medical College, Shangrao, Jiangxi China; 3 Present address: School of Nursing, Gannan Medical University, Ganzhou, Jiangxi China

## Abstract

**Background:**

The prevalence of hypertension is increasing worldwide. Hypertension self-management usually involves the application and consideration of oral, written, or quantitative information. Hypertensive patients in China have limited high blood pressure health literacy (HBP-HL), which may lead to poorer clinical outcomes. This study aims to determine the feasibility and effectiveness of an interactive pictorial health education program based on behavior change wheel (BCW) theory and its effect on HBP-HL, self-efficacy, self-management ability, and health-related quality of life (HRQOL) in hypertensive patients with low HBP-HL.

**Methods:**

This study is a randomized controlled trial (RCT). One of the municipal districts in Huzhou, China, will be randomly selected, and two communities with similar conditions within this district will be screened and selected. A total of 120 hypertensive patients aged 18 years and older will be recruited from these two community settings. One of the communities will be randomly allocated to an interactive pictorial health education program conducted by a comprehensive health literacy strategy that includes (i) training participants in effective health communication skills that address issues encountered in seeking medical care and (ii) the use of self-developed interactive pictorial hypertension education to improve patient understanding and behaviors versus a control group (routine community lecture health education only). The primary outcome measure is HBP-HL. Secondary outcomes are self-efficacy, self-management ability, HRQOL, social support, and improvement in blood pressure. Outcomes will be collected at 6, 9, and 12 months from trial entry.

**Discussion:**

The strengths of this study are the establishment of a new health management program for hypertensive patients that closely combines BCW theory and health literacy. This trial has the potential to improve HBP-HL in hypertensive Chinese patients with low health literacy to improve the self-management of hypertension and help control blood pressure.

**Trial registration:**

Clinical Trials.gov Protocol Registration and Results System ID NCT04327102. Prospectively registered on February 29, 2020

## Strengths and limitations of this study

- The main strength of the study lies in establishing a new health management program for hypertensive patients combining BCW theory and HBP-HL closely to better address the problem under study.
- Further strengths are that we will match the intervention and control cases in order to reduce bias and confounding factors as much as possible as well as the fact that data are collected in a naturalistic situation.
- One limitation is that the trial is not double-blinded and will be implemented in only two communities.
- Another possible limitation of this study is the short 9-month intervention and 3-month follow-up intervention, which may not be long enough to observe the effect of the intervention on the occurrence of hypertension complications.

## Background

As one of the most common cardiovascular diseases in the world, hypertension has been proven to be a primary and independent risk factor for coronary heart disease, heart failure, stroke, and other cardiovascular and cerebrovascular diseases [1]. At present, complications of hypertension are the leading cause of death globally and are extremely common in many low- or middle-income countries, causing approximately 10.4 million deaths worldwide each year [2]. According to the American Heart Association (AHA), the number of deaths caused by hypertension and its complications will increase to more than 23.6 million by 2030 [3]. Research has shown that the number of hypertensive patients in China is 245 million (the prevalence rate is 23.2%), and it is still growing [4]. Due to the large number of hypertensive patients and the lifelong health care the condition entails, strengthening the health management of hypertension worldwide is urgently needed.

Health-related quality of life (HRQOL) is one of the main indicators used to evaluate the health management of hypertension [5]. Meanwhile, multiple studies have shown that health literacy is one of the main influencing factors for HRQOL in hypertensive patients and is positively correlated with HRQOL [6–8]. A completed cross-sectional investigation by our team confirmed that health literacy was the key influencing factor of HRQOL in hypertensive patients from Huzhou. In addition, health literacy is an independent predictor of blood pressure control and can predict patients' health outcomes better than sociodemographic data (age, income, occupation, education level and race) [9–11].

The American Institute of Medicine defines health literacy as “individuals’ ability to obtain, understand, and process basic health information and services needed to make appropriate health-related decisions” [12]. Nutbeam describes it as “interactive, functional and critical health literacy, reflecting a comprehensive ability” [13]. The literacy of chronic disease prevention and treatment was 15.71% among Chinese residents in 2017, an increase of 6.64% compared with 2012, but still at a relatively low level [14]. A 10-region prospective study in China showed that the prevalence of hypertension was highest in Zhejiang (44.4%) [14]. Only 6.83% of hypertensive patients have adequate health literacy [15]. Patients with inadequate health literacy lack disease prevention and health care knowledge and cannot search for information on health and self-management [16, 17]. They ultimately lose the best opportunity for prevention and treatment with an increased incidence of complications. Although their medical utilization is greatly reduced, the medical costs sharply increase [18, 19]. Studies show that people with low health literacy are 1.5–3 times more likely to have adverse health outcomes than those with high health literacy [20]. However, it is remarkable that intervention for patients with low health literacy can effectively improve their health status [9]. One of the most promising measures to achieve national health is to strengthen health education, improve health literacy, and enable patients with chronic diseases to learn related abilities of chronic disease management [21, 22].

It has been proven that providing materials or information on communication skills for people with low literacy can improve comprehension for patients with both low and high literacy [20, 23]. The “Conversation Map” is a very practical interactive health education tool launched by the International Diabetes Federation [24]. As an educational medium, pictures can lead patients to discuss with each other and realize a heuristic approach to answering questions. Different kinds of pictures have been adapted in health education for diseases such as stroke, bronchial asthma, and chronic heart failure [25, 26], which improves acceptance and meets the needs of patients with low health literacy. To date, very few studies have been specifically implemented to develop pictures and intervention strategies suitable for hypertensive patients with low health literacy to improve their health literacy. Addressing literacy through improved educational materials and intervention strategies is a potentially successful strategy. It is an innovative method to optimize patients’ understanding, improve their health literacy and HRQOL, and enhance their self-efficacy and self-management behaviors.

**Fig. 1:**
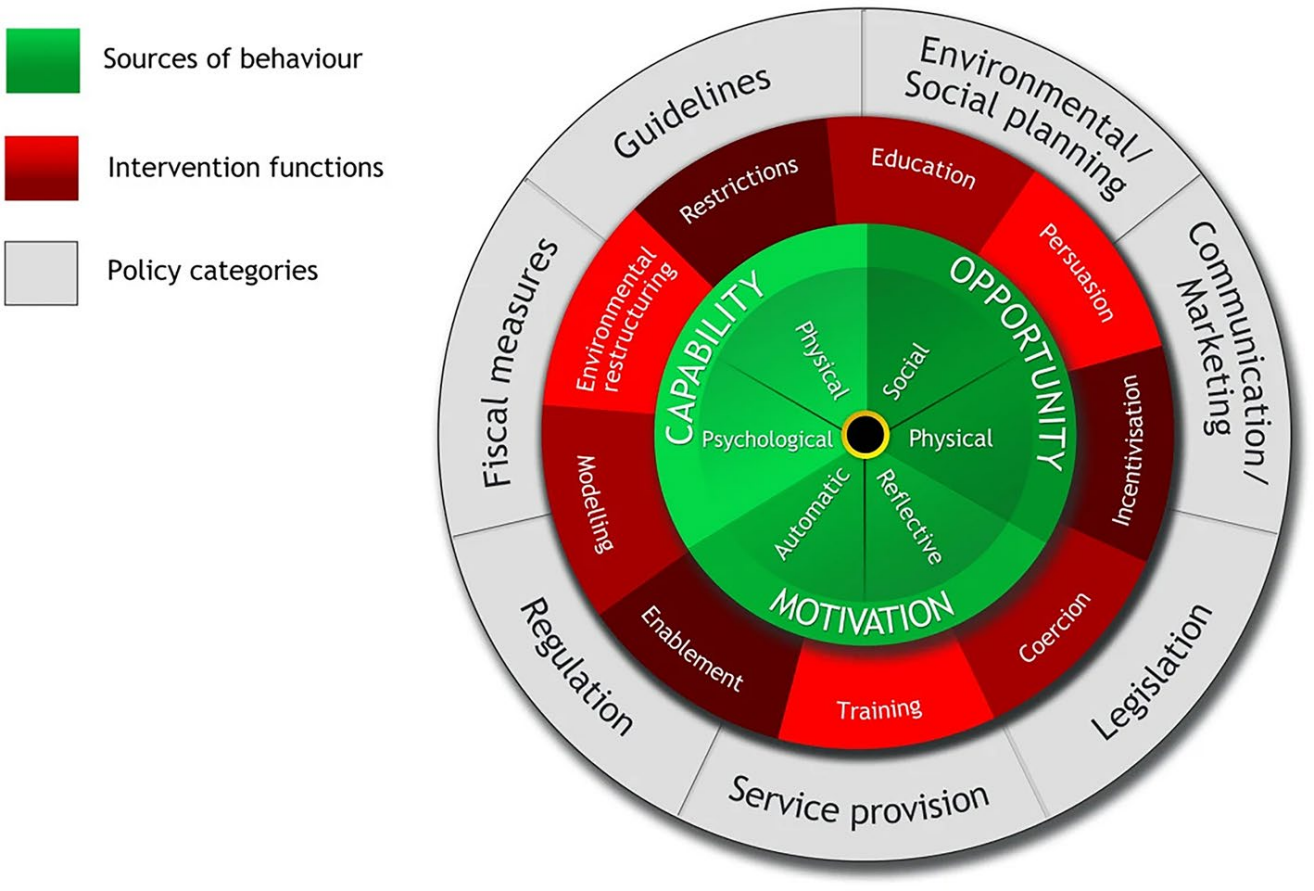
The behavior change wheel consists of three layers. The center layer is composed of the Capability, Opportunity, and Motivation model. The second layer is composed of nine intervention functions which are the general methods by which interventions might change behavior. Examples are “education” and “persuasion.” The topmost layer of the wheel itemizes the policy categories that can be used to support the implement of these functions

Studies show that intervention strategies based on the principle of behavior change are more robust, efficient, and practical [27, 28]. The BCW theoretical model (Fig. 1) [29] is a comprehensive behavior change system rooted in an integration of 19 behavior change frameworks. The wheel consists of three layers. At the center, the Capability, Opportunity, and Motivation model includes subjective factors (physical and psychological capability, automatic and reflective motivation) and objective factors (physical and social opportunity) and identifies the conditions required for behavior to occur and provides a method for formulating corresponding health management plans to promote healthy behavior change. The second layer is composed of nine intervention functions (Fig. 1), which are the general methods by which interventions might change behavior. The topmost layer of the wheel itemizes the policy categories that can be used to support the implementation of these functions.

To address this problem, we intend to develop an interactive pictorial health education program based on the nine intervention functions of BCW theory, which is targeted to improve the health literacy and self-management capacity for hypertensive patients with low health literacy.

Our study attempts to answer two primary questions:

(i) Is it feasible to use the interactive pictorial health education program based on BCW theory for application in the community setting? We hypothesize that hypertensive patients with low health literacy would be willing to participate in this program and would have high compliance, and there would be no adverse events occurring during the intervention.
(ii) What are the impacts of interactive pictorial health education programs based on BCW theory on hypertensive patients with low health literacy? We hypothesize that participants receiving BCW-based interactive pictorial health education program interventions compared with a routine community lecture health education only group will demonstrate an improvement in HBP-HL, HRQOL, self-management ability, and self-efficacy and a reduction in blood pressure.

## Methods

### Study design

**Fig. 2:**
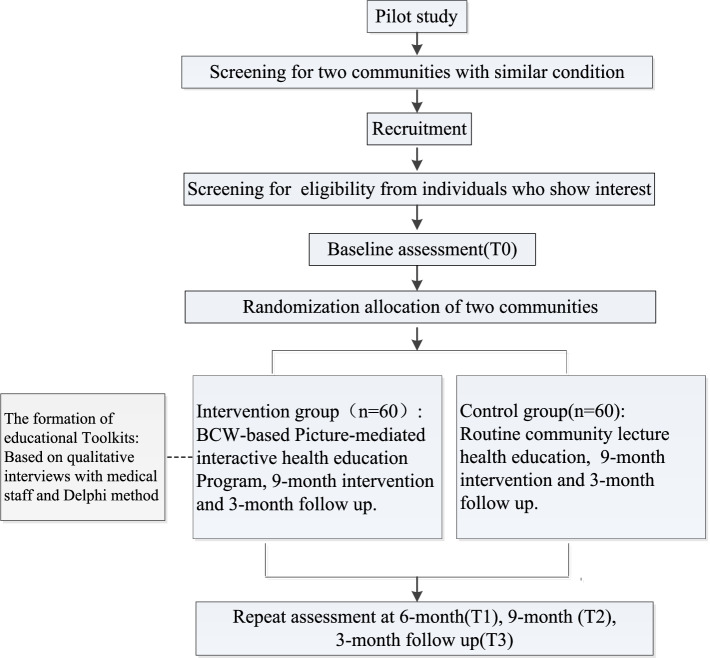
Overview of the study procedure

This is an RCT (Fig. 2) with a 9-month intervention and a 3-month follow-up. This program will consist of two arms: the intervention group (BCW-based interactive pictorial health education program) and the control group (routine community lecture health education). Prior to the trial, we completed the final draft of the BCW-based interactive pictorial health education program through medical staff and patient interviews and the Delphi method. A pre-experiment was conducted to assess the acceptability and feasibility of the intervention and research procedures in hypertensive patients with low health literacy in Huzhou, Zhejiang, China. We obtained informed consent from each study participant. The results of the two studies have been used to inform the development of the forthcoming trial.

### Setting

The participants will be recruited from the community settings in Huzhou, Zhejiang Province, China. First, one of the municipal districts in Huzhou will be randomly selected, and then two communities with similar conditions within the district will be screened and selected. Finally, the two communities will be randomly allocated to the intervention group and the control group. Subjects meeting the inclusion criteria will be enrolled into the intervention group and the control group after informed consent is obtained. In addition, we will contact community health providers to participate in the study to ensure the cooperation of participants**.**

### Recruitment and study population

Our researchers will give out recruitment leaflets in the two selected communities. Health care providers in each community will also help introduce eligible individuals to our study. Three trained researchers will participate in an in-person interview to screen individuals who show interest for eligibility. All participants will be fully informed of the study protocol first and will only be included in this study upon signing informed consent before baseline measurements are taken. They will receive a gift for the completion of each session and each data collection stage. In addition, those who have a severe mental disorder or cognitive impairment at present, or who experience adverse events during the intervention or follow-up period will be withdrawn from the study. Detailed inclusion and exclusion criteria are as follows.

Inclusion criteria:

- Aged ≥ 18 years and have lived in Huzhou for at least half a year
- Diagnosed as hypertensive according to the 2018 Chinese Guidelines for the Prevention and Treatment of Hypertension, systolic blood pressure (SBP) ≥ 140 mmHg and/or diastolic blood pressure (DBP) ≥ 90 mmHg
- Chinese High Blood Pressure-Health Literacy Scale (C-HBP-HL) score < 40 points
- No major stress events (e.g., the death of relatives) within the past 6 months
- Able to communicate in Mandarin, voluntarily participating in the study, and signing the informed consent of research intention
- At least one modifiable cardiovascular risk factor

Exclusion criteria:

- Any individuals with secondary hypertension
- Severe mental disorder or cognitive impairment

### Randomization and blinding

#### Sequence generation

After completing the screening of the two communities with similar size, educational background and economic income, randomization will be performed at the level of the community after recruitment is completed. One community will be randomized to receive the intervention, and the other community will be randomized to the control group by throwing dice. The randomization process will be completed by a third party unrelated to the study. Only after the subjects have completed the baseline data collection, the allocation personnel can inform the researchers of the allocation results.

#### Allocation concealment

Participants and the trial coordinator will not be blinded to their group allocation; however, the data collectors and the data analyst will be blinded. The codes will be broken following statistical analysis.

#### Sample size calculation

The Gpower3.1.9.2 software was used to conduct the sample size calculation. The sample size was estimated by power analysis using the Chinese high blood pressure-health literacy scale for the evaluation of HBP-HL as a primary outcome measure of the study. Cohen’s *d* and the effect sizes were calculated for between-group differences of means of the level of HBP-HL using an independent-groups pre-test and post-test design. From the evidence of our pilot study, the HBP-HL score of the intervention group was 32.09 ± 4.89 points, and that of the control group was 27.80 ± 4.78 points, which led to an estimate effect size as 0.405. The analysis, with a two-sided test using *β* = 0.2, *α* = 0.05, two sample *t-test*, and repeated ANOVA, requires a total sample size of 108 participants. Considering a 10% dropout rate, our final sample size comes to 120 participants (60 participants for both the intervention and control groups).

#### Intervention

Participants in each group will be divided into 3 teams of 15–20 people. Intensive intervention will be carried out for 9 months and twice a month in the first 2 months. During the other months, the intervention will be conducted once a month. Later, 3 months of follow-up will be conducted over telephone once a month and hypertension-related knowledge and videos (Putonghua and Huzhou dialect version) will be sent to the WeChat group once a week. Intermediary data will be collected at the 6th and 9th months to obtain timely feedback, to correct deviations, and to monitor and ensure the successful implementation of the intervention.

#### Intervention group

Interactive pictorial health education content (see Table 1) is drawn up according to 9 intervention functions of BCW theory that may stimulate behavioral change through several mechanisms of action.

**Table 1:** Description of intervention content based on BCW theory

Intervention component	Intervention function	Mechanisms of action (COM)	Intervention time
**•**Explain the basic health knowledge of hypertension, help patients to acquire a correct concept of hypertension	Education	Psy C, Ref M	Week 1 of the first month
•Teach blood pressure monitoring skills, train patients to communicate with medical staff effectively	Training	Psy C, Phys C	Week 3 of the first month
•Recognize the hypertension complications, teach and encourage to set the goal of behavior change by themselves	Persuasion Realization	Psy C, Phys C, Ref M, Auto M, Soc O, Phys O	Week 1 of the second month
•Help patients understand the prevention measures of hypertension complications (medicine, diet)	Incentivisation	Ref M, Auto M	Week 3 of the second month
•Help patients understand the prevention of hypertension complications (exercise, weight management, sleep and psychological regulation)	Incentivisation	Ref M, Auto M	3th months
•Review the knowledge of complications of hypertension and feedback on behavior	Environment reconstructing	Soc O, Phys O, Auto M	4th months
•Review the preventive measures for complications of hypertension. Group instruction for patients with different health literacy problems	Environment reconstructing	Soc O, Phys O, Auto M	5th months
•Review blood pressure monitoring knowledge + peer knowledge sharing	Modeling	Auto M	6th months
•Review blood pressure monitoring knowledge + peer knowledge sharing	Modeling	Auto M	7th months
•Review blood pressure monitoring knowledge + peer knowledge sharing	Modeling	Auto M	8th months
•Review hypertension related knowledge	Education	Psy C, Ref M	9th months
• Follow-up with telephone and Wechat app	Education Environment reconstructing	Psy C, Ref M, Auto M, Soc O, Phys O	10th, 11th, and 12th months

A pictorial map will be used to visualize the content of health education and to guide the steps of the content, which is more acceptable for hypertensive patients with low HBP-HL. On the pictorial map, the size of which is 1 × 1.2 m, the text is written in a large, bold font to accommodate low functional health literacy levels and eyesight. The navigation arrows on the map indicate the steps of the intervention.

The educational content was developed by researchers based on a review of previous related research and programs and focused on medication, diet, exercise and weight management, sleep and psychological regulation, and knowledge of blood pressure monitoring and complications. Five experts evaluated the content of the health education program. The expert group consisted of cardiology, nursing, psychology, and pharmacology professors. The participants will use drawing implements to mark their progression through the educational content. The pictorial map will guide interactive discussions, which will facilitate the participants’ ability to acquire knowledge about hypertension. At the beginning of each intervention, educators will organize all participants to sit around the pictorial map in a U-shape. Each theme on the map comes with a set of questions that will be used as a framework by the facilitator to lead the discussion. In addition, the participants are encouraged to find the elements on the picture according to the theme and questions, guide the topic of development gradually through the corresponding content, and discuss with each other to deepen their understanding and memory. For example, the first question when using the map is to ask if they know the complications of hypertension. Ultimately, educators will guide everyone to review all the content discussed in the activity and answer the questions that they have not discussed yet to help participants sort out and consolidate the knowledge gained during the session.

#### Control group

The control group will only receive routine community lecture health education sessions, 9 months of intervention, and 3 months of follow-up.

### Outcomes and measuring instruments

#### Sociodemographic questionnaire

The questionnaire is a self-administered survey including age, gender, education, occupation, financial status, marital status, living condition, insurance status, smoking and alcohol status, history of hypertension, family history of hypertension, types of blood pressure medications, self-test blood pressure status, and complications.

#### Feasibility

We will calculate the proportion of participants completing the intervention. Attendance will be examined as the proportion of participants who attend 9/11 sessions and the average number of sessions attended. At the end of each intervention session, a 5-point Likert scale (not at all to very much) will be used to rate all participants to gauge their level of satisfaction with the project and their mental and social engagement. At the 3-month follow-up assessment, participants will indicate their level of interest in participating in the project in the future and whether they would recommend it to others.

#### Outcome evaluation scales

##### Chinese high blood pressure-health literacy scale (C-HBP-HLS) [30]

This scale is divided into 5 dimensions (Print Health Literacy, Medication Label, Understanding Ability, Newest Vital Sign Test, and Avoiding Food Allergy) consisting of 15 items, which was translated into Chinese and validated by Zhang Q et al. in 2016. According to the test of functional health literacy in adults (TOFHLA) scoring system, the final total score range is 0–60 points, and the higher the score is, the higher the HL level is. Furthermore, according to the TOFHLA classification criteria, the final score of the HBP-HL scale is divided into 3 grades: a total score < 32 points reflects a lack of HBP-HL; a total score ranging from 32 to 40 points indicates an intermediate level of HBP-HL; and a total score ≥ 40 points represents a high level of HBP-HL. The Cronbach’s *α* coefficient of the scale was 0.78, and the retest reliability was 0.96.

##### Hypertensive patients’ self-management behavior rating scale (HPSMBRS) [31]

This scale was developed and validated by F Zhao, Q et al. in 2012 and consists of 33 items in 6 dimensions (medication management, condition monitoring, diet management, exercise management, work and rest management, emotional management). The final total score ranges from 33 to 165 points. Higher scores reflect a better self-management behavior level in hypertensive patients. The Cronbach’s *α* coefficient of the scale was 0.914, and the content validity was 0.910.

##### Chinese SF-36 health survey [32]

The SF-36 has nine evaluation items, including physiological function, physiological function, physical pain, general health status, social function, emotional function, mental health, vitality, and one self-assessment of health changes. The total score is 145. The higher the score, the better the health-related quality of life.

##### Chronic disease self-management program (CDSMP) [33]

This program was developed by Lorig of Stanford University in 2001 during research on the self-management behavior of patients with chronic diseases. The scale includes 6 items using a 1–10 grade scoring method: level 1 means no confidence, level 10 means full confidence, the total average score of 6 items is scored, and the total score of the scale is 1–10, including 2 dimensions (symptom management self-efficacy, disease common management self-efficacy). The higher the score, the higher the level of self-efficacy. The total score of self-efficacy can be divided into three levels (low level: total score < 5, medium level: 5 ≤ total score < 7, high level: total score ≥ 7).

##### Perceived social support scale [34]

The scale is divided into 3 dimensions and 12 items, mainly including family support, friend support and other support. The scale adopts the scoring method from 1 to 7, and the higher the score is, the higher the social support level of participants.

### Clinical measurements

Blood pressure will be measured by the same surveyor with an Omron HEM-7211 electronic sphygmomanometer after the patient has rested quietly for at least 5 min. The measurement will be repeated at intervals of at least 5 min, and the average of the two readings will be recorded.

Anthropometric measurements, including height (cm), weight (kg), neck circumference (NC) (cm), waist circumference (WC) (cm), and hip circumference (cm), will be measured by researchers for all participants in lightweight clothing and bare feet. Body weight will be obtained using an Omron HBF-214 electronic scale, height will be reported by participants, and NC, WC, and hip circumference will be measured using a clinical tape measure. The parameters will be measured and recorded to the nearest 0.1 cm or 0.1 kg. Body mass index (BMI) is referred to as weight (kg)/height (m)^2^. According to the Working Group on Obesity in China, BMI is divided into normal weight (18.5 ≤ BMI < 24 kg/m^2^), overweight (24 ≤ BMI < 28 kg/m^2^), and obesity (BMI ≥ 28 kg/m^2^) [35]. NC is determined by measuring the horizontal circumference of the neck between the lower margin of the thyroid cartilage (Adam's apple) and the upper margin of the 7th cervical vertebra. WC is measured at the midpoint between the lateral anterior superior iliac spine and the lower border of the tenth rib. Using criteria from the WHO for the Chinese population, a WC ≥ 90 cm for men and ≥ 80 cm for women is considered abdominal obesity [36].

Measurements will be taken at baseline and at 6, 9, and 12 months from trial entry **(**the detailed timeline is shown in Table 2). To decrease the dropout rate, some retention strategies will be carried out: (1) collecting contact information of participants and their family members, (2) distributing a gift for every participant at each event, and (3) providing a quiet and comfortable environment for the intervention. In addition, the measurements will be collected by designated and appropriately trained and blinded assessors.

**Table 2:** Summary of study assessments and timelines

Questionnaire items		Time point				
		Baseline T0	Randomized Allocation	The 6th month of intervention T1	The 9th month of intervention T2	Follow-up 3 months T3
Enrollment Eligibility screening Informed consent Screening						
Primary outcomes	C-HBP-HLS	√		√	√	√
Secondary outcomes	CDSMP	√		√	√	√
	HPSMBRS	√		√	√	√
	Perceived social support scale	√		√	√	√
	Chinese SF-36 Health Survey	√		√	√	√
	SBP	√		√	√	√
	NBP	√		√	√	√
	BMI	√		√	√	√
	NC	√		√	√	√
	WC	√		√	√	√
	hip perimeter	√		√	√	√
Sociodemographic data		√				

### Data management

Data from the two stages of the study will be stored in a password-protected computer folder in the researcher’s office, where no one else is allowed to enter. Postgraduate students will be responsible for collecting, coding, analyzing, and recording the data; however, the Supervising Committee composed of community health care providers who have no competing interests with the sponsor and who will monitor the whole process. Only the research team will have access to the data.

### Statistical analysis

The SPSS 23.0 software will be used for data processing and statistical analysis. Descriptive statistics will be used to process sociodemographic data, of which observable variables are presented as the means ± standard deviations for continuous variables and frequencies and percentages for categorical variables. Exploratory analysis will be applied to examine the distribution of data. Baseline data comparisons between the two groups will be conducted using the chi–square (*χ*^2^) test (for categorical variables) and *t-test* or Wilcoxon rank-sum test (for continuous variables). 2(group)× 3(time) ANOVA is used for data with a normal distribution and homogeneity of variance, and a generalized estimation equation is used for data with a nonnormal distribution to determine the main effect of intervention factors, time factors, and interaction between group factors and time factors. Simple effects will be further analyzed when there are interaction effects. A two-tailed *P* < 0.05 will be considered statistically significant.

### Dropouts and missing data

Reasons for dropouts will be documented and reported. If patients are alive and missing at random, we will use multiple imputation methods based on ten imputed datasets with multiple imputation by chained equations [37].

### Quality control

In collaboration with community health care providers before the trial entry, health care providers in each community are responsible for monitoring patient recruitment, consent, and collection of measurements. All researchers will be trained uniformly. To prevent bias, the data will be collected with unified measurements and analytical instruments by designated personnel (not the researcher himself) who are not clearly conducting the grouping. As soon as a questionnaire is completed, it will be strictly screened, and if there is any missing data, it will be completed in time. All data collection will be completed, encoded, and inputted by two people, and questionnaires with obvious errors will be removed. Epidata3.1 will be used to establish the database, randomly check and review it at a rate of 10%, and detect errors and correct them in a timely manner.

## Discussion

This study aims to identify whether a BCW-based interactive pictorial health education program can better improve HBP-HL and HRQOL for hypertensive patients with low health literacy. The research method of heuristic questioning instead of didactic education will also demonstrate that this type of intervention can effectively mobilize the subjective initiative of hypertensive patients and provide a scientific and effective method for hypertension health management in the community. Furthermore, this study aims to provide new ideas and methods for hypertensive patients to effectively control their blood pressure and improve their HRQOL.

We hypothesize that participants in the intervention group will have effective improvements in HBP-HL, blood pressure, HRQOL, self-management ability, self-efficacy, and other patient outcomes essential in improving hypertension care when compared to the control group patients. We also hypothesize that health care providers in the intervention group will report improved health communication skills and increased knowledge and satisfaction compared to providers in the control group.

To date, very few studies combining BCW theory with interactive health education with “picture dialogue” have been designed for hypertensive Chinese patients. In light of the status quo that the prevalence of hypertension in Zhejiang Province is high but health literacy related to chronic diseases is low, if the study hypotheses are verified, a BCW-based interactive pictorial health education program could be more widely spread and applied in hypertension health care, limiting medical costs due to hypertension complications.

The key benefit of this study will be the development and application of BCW-based interactive pictorial intervention materials to promote HBP-HL in hypertensive patients with low health literacy in Huzhou. The materials developed for the project will complement and expand upon existing health education materials, such as those available from community service centers, which will focus on how to help people with low health literacy improve their health literacy levels and change unhealthy health behaviors rather than just providing information. If this project is proven effective, health care professionals and providers in community health care centers will be provided with a validated theory-based interactive pictorial health education program and will be prompted to increase its application in hypertensive patients with low health literacy.

### Dissemination policy

We will present our findings in peer-reviewed journal articles. Attendees will be informed of consent for publication to ensure their identities will not be disclosed. All methods will be carried out in accordance with SPIRIT guidelines and regulations.

### Protocol amendments

If there are any issues that may affect the trial progress and participants’ interests or safety, the protocol needs to be formally modified. These amendments need to be agreed by the Supervising Committee and approved by the Ethics Committee prior to implementation.

### Ancillary and post-trial care

Not applicable.

## Trial status

This study is ongoing. Recruitment began in July 2021 and will finish in May 2022.

## Data Availability

This is a study protocol; therefore, data sharing is not applicable, as no data are generated or analyzed.

## Abbreviations

HRQOL: Health-related quality of life
RCT: Randomized controlled trial
AHA: American Heart Association
BCW: Behavior change wheel
HBP-HL: High blood pressure health literacy
C-HBP-HLS: Chinese High Blood Pressure-Health Literacy Scale
HPSMBRS: Hypertensive Patients’ Self-Management Behavior Rating Scale
CDSMP: Chronic Disease Self-Management Program
